# Knowledge and Lifestyle Behaviors Related to COVID-19 Pandemic in People over 65 Years Old from Southern Italy

**DOI:** 10.3390/ijerph182010872

**Published:** 2021-10-16

**Authors:** Francesca Gallè, Elita Anna Sabella, Paolo Roma, Stefano Ferracuti, Giovanna Da Molin, Giusy Diella, Maria Teresa Montagna, Giovanni Battista Orsi, Giorgio Liguori, Christian Napoli

**Affiliations:** 1 Department of Movement Sciences and Wellbeing, University of Naples “Parthenope”, Via Medina n. 40, 80133 Naples, Italy; giorgio.liguori@uniparthenope.it; 2 Inter-University Research Centre “Population, Environment and Health”, University of Bari “Aldo Moro”, Piazza Umberto I, 1, 70121 Bari, Italy; elita.sabella@uniba.it (E.A.S.); giovanna.damolin@uniba.it (G.D.M.); 3 Department of Human Neurosciences, “Sapienza” University of Rome, Piazzale Aldo Moro 5, 00185 Rome, Italy; paolo.roma@uniroma1.it (P.R.); stefano.ferracuti@uniroma1.it (S.F.); 4 Department of Biomedical Sciences and Human Oncology, University of Bari Aldo Moro, Piazza G. Cesare 11, 70124 Bari, Italy; giusy.diella@uniba.it (G.D.); mariateresa.montagna@uniba.it (M.T.M.); 5 Department of Public Health and Infectious Diseases, “Sapienza” University of Rome, Piazzale Aldo Moro 5, 00185 Rome, Italy; giovanni.orsi@uniroma1.it; 6 Department of Medical Surgical Sciences and Translational Medicine, “Sapienza” University of Rome, Via di Grottarossa 1035/1039, 00189 Rome, Italy; christian.napoli@uniroma1.it

**Keywords:** COVID-19, knowledge, lifestyle, elderly

## Abstract

Background: Control measures adopted during the COVID-19 pandemic had a considerable impact on human daily life and lifestyles. Adherence to the recommended measures is influenced by knowledge and attitudes towards the disease. This cross-sectional study aimed to assess the level of knowledge regarding COVID-19, the related control measures, and lifestyle behaviors adopted during the pandemic in a sample of elderly Italian people. Methods: A web-based questionnaire investigating socio-demographic characteristics, knowledge of COVID-19, the related preventive measures, and lifestyle changes that occurred during the pandemic, were distributed to ≥65 years old people living in South Italy. Results: A satisfactory level of knowledge about COVID-19 and the related control measures has been found in the enrolled sample. However, a decrease in physical activity, as well as worsening sleeping and dietary habits, were found in roughly 60% of participants. Females were impacted greater by these lifestyle changes. Conclusions: Participants in this study showed a good level of knowledge regarding COVID-19 and its prevention. Nevertheless, they reported an increase in unhealthy habits that may have important health consequences in the long term and should be addressed by public health interventions targeted at older people.

## 1. Introduction

During the first months of 2020, a novel coronavirus, previously detected in China, arrived in Italy, causing a severe acute respiratory disease (SARS-CoV-2) [1]. The virus spread rapidly across the country in consecutive waves, causing thousands of hospitalizations and deaths [1,2], with an uneven impact throughout the regions of Italy, especially during the first wave [3]. Northern regions saw a higher infection rate (up to 763 cases per 100,000 inhabitants in Valle D’Aosta); lower values were reported in central regions (up to 389 per 100,000 inhabitants in Marche) and these figures were even lower in Southern regions (up to 174 per 100,000 inhabitants in Abruzzo) [3]. Older adults were the main victims, and a correlation between mortality in older adults and the number of people hosted in long-term care facilities has been observed [3]. However, the incidence rate of COVID-19, outlined above, does not fully explain the differences in mortality among older adults in different regions. Moreover, the percentage of deaths per population was lower in the greener Mediterranean regions compared to the northern regions with low forest coverage [4]. This evidence can likely be explained by sea proximity, a mild climate, a Mediterranean diet, and the abundance of non-deciduous Mediterranean plants that emit immunomodulatory and antiviral compounds [4].

The epidemiological situation forced the Italian government to adopt a series of control measures aimed at hindering the circulation of the virus in line with the recommendations of the World Health Organization (WHO), such as social distancing, the obligatory use of facial masks [5], and contact tracing for SARS-CoV-2, given the demonstrated effectiveness of screening for infectious diseases in the at-risk population [6,7,8]. In order to further limit transmission, measures to restrict movement were also imposed. A complete lockdown, with the prohibition of non-essential activities, was imposed from March to May 2020; subsequently, other restrictions regarding meetings and events were established in relation to the ongoing epidemiological situation [5,9,10].

These measures had a considerable impact on several aspects of daily human life and social activities, with consequences on lifestyles, health, and well-being [11,12,13]. However, adherence to these measures was fundamental before and during the immunization campaign, which commenced in Italy during December 2020 and aimed to contain and reduce the epidemic. Evidence shows that adherence to the recommended measures was influenced by knowledge, attitudes, and practices towards the disease [13,14,15].

Several studies have been performed in Italy to assess the awareness of COVID-19, and the related preventative measures, in the general population and in specific working categories [16,17,18,19]. With some exceptions, these studies reported good levels of knowledge about the pandemic. As for behaviors, physical activity (PA) decreased notably during the lockdown, especially among already inactive people [19,20].

To the authors’ knowledge, no study has been performed to investigate awareness and behaviors related to the COVID-19 pandemic among the Italian elderly. Considering that this age group is highly interested in the direct and indirect negative consequences of the disease, it is fundamental to evaluate these aspects in this specific population.

Therefore, the present cross-sectional study was aimed to assess (1) the level of knowledge regarding COVID-19 etiology, transmission, and the related control measures, and (2) the changes that occurred during the pandemic to the lifestyles and behaviors of a sample of the Italian elderly.

## 2. Materials and Methods

### 2.1. Setting and Participants

This cross-sectional study was carried on from June to August 2021 among ≥65 years old Italian people. The study was conducted in the Apulia Region, which covers a large part of southern Italy with a total surface area of around 20,000 km^2^ and hosts roughly 1 million inhabitants over 65 years old. Subjects were chosen via convenience sampling, including those who participate in religious, recreational or cultural associations or live in facilities that host self-sufficient elderly inhabitants. According to the stratification of the regional population, people aged over 65 years old, living in each of the 6 provinces of the region, were invited to voluntarily participate in the survey by responding to an online questionnaire [21]. The choice of an online questionnaire was made due to the need to reduce contact between people and minimize paper usage. The link to the web-based questionnaire was sent, via social media or by email, directly to the elderly, or through a reference person working in the housing facilities. In both cases, the online questionnaire was asked to be disseminated to the maximum number of people possible.

The Apulian target population is made up of 891,842 people aged over 65; therefore, a sample of at least 384 individuals was estimated to evaluate the selected variables, assuming a 5% margin of error, and a response proportion of 50% with a 95% confidence level [22].

This study followed the rules stated by the World Medical Association Declaration of Helsinki. The study was approved by the Scientific Institutional Review Board of the Italian Inter University Research Centre “Population, environment and health” (n. 0530_2021, dated 23 June 2021).

### 2.2. Questionnaire

A questionnaire comprising of three sections, written in Italian, was electronically distributed to participants. In order to not exclude older people with limited access to digital media or the internet, a paper questionnaire was also provided upon request.

The first section of the questionnaire aimed to collect socio-demographic information such as gender, age, educational level (none/elementary/middle/high/university degree), and place of residence (community/institution). Participants were also asked to self-report their height and weight values in order to calculate their body mass index (BMI) and related weight status (underweight/normal/weight/overweight/obese) according to the WHO classification [23]. Interviewees were also asked to refer their weight before the pandemic in order to highlight possible changes. These items were designed on the basis of a previous study performed in the same population [24] and the opinions of a panel of experts composed of one demographer, one epidemiologist, and one nutritionist.

The second part included questions about knowledge related to SARS-CoV-2 transmission and its preventive measures. This part was structured by adapting a questionnaire used in previous studies to the specified age group [19]. The modified items included in this section were stated by a panel of experts, including one public health expert, one epidemiologist, and one biologist expert in infectious diseases.

Participants were asked to:

1. judge if the new epidemic may be considered similar to that of the seasonal flu (yes/no);
2. identify which was the total number of deaths in Italy as a result of COVID-19 at the time of the survey (1000/10,000/more than 100,000);
3. identify the cause of the new epidemic as a virus, a bacterium, or a parasite;
4. identify the target population of COVID-19 (only older people/only children/only healthcare personnel/all);
5. refer to the main transmission route (air/water/food/blood transfusion);
6. report the most effective protective measures (handwashing/facial mask/protective glasses/washing fruit and vegetables/disinfection of surfaces/avoid close contact/vaccine);
7. reply about the availability of an effective drug for COVID-19 treatment (yes/no).

The third section was aimed at investigating possible changes in the lifestyle of elderly people over the course of the pandemic. The questions concerned current dietary habits (improved/worsened/same as before), smoking (less than before or stopped/more than before or started/smoking as before/not smoking as before), and PA (increased/decreased/same as before). These questions, used in the previous study, were adjusted to fit the specific target population [19]. In addition, participants were also asked to refer to their sleep (improved/worsened/same as before) and walking habits, for duty or pleasure, during the pandemic in comparison to the pre-pandemic period (increased/decreased/same as before). This section was curated by a panel of experts, including one epidemiologist, one psychologist and one expert in movement sciences.

Participants were informed that the submission of the questionnaire implied informed consent. The data were kept confidential, and the results did not allow the personal identification of the respondents. A preliminary study involving 54 people over 65 years old was carried out in order to test the questionnaire’s validity (data not published). The Intelligibility of the questions was evaluated by asking the enrolled sample to assign a 7-point score (from 1: not meaningful to 7: very meaningful) to each question. Furthermore, in order to guarantee answer variability, the original questionnaire was modified in the preliminary study: 10 further questions (FQ), reporting grammatical and/or semantic errors, were added to the original questions (OQ). The OQ reported a mean score of >6 for each question; the FQ reported a mean score of ≤1. These data confirmed that the content of the questionnaire was clear to the readers. Cronbach’s alpha (internal consistency coefficient) was used to test the reliability index for both the preliminary and original study [25,26]. The alpha values showed a good level of reliability (0.85 and 0.81, respectively) [27].

### 2.3. Statistical Analyses

Respondents’ socio-demographic, anthropometric and behavioral characteristics were the object of a descriptive analysis. The mean age ± standard deviation (SD) was evaluated. Other variables and answers were analyzed as the number and percentage of the respondents. Comparison between genders was performed via the chi-squared test. A Kendall’s correlation analysis was carried out to highlight possible relationships between socio-demographic variables, the level of COVID-19 knowledge, and lifestyles adopted during the pandemic. In particular, age, gender (0 = male, 1 = female), level of education (0 = elementary, 1 = middle, 2 = high school, 3 = degree), and place of residence (0 = community, 1 = institution) were included as demographic variables. The level of knowledge was expressed as the total number of correct answers (range 0–7); multiple answer questions were considered correct when at least one correct answer was chosen by the respondent. Lifestyle and communication variables were categorized as worsened (0), maintained (1), and improved (2).

A *p*-value of 0.05 was assumed as a significance level. The software IBM SPSS version 27 for Windows (IBM Corp., Armonk, NY, USA) was used for the analyses.

## 3. Results

A total of 1209 questionnaires were collected, of which 1041 (86.1%) answered completely, correctly fulfilled, and were considered for the analysis. Table 1 shows the main characteristics of the sample.

Table 2 reports the answers given by respondents about COVID-19 knowledge.

More than 80% of the sample distinguished the COVID-19 epidemic from that of seasonal flu and correctly identified the current number of deaths, the cause, the target, the main transmission route of the disease, and the most effective preventive measures. A total of 75% reported that an effective drugs against COVID-19 is not available.

Table 3 reports the answers related to participants’ lifestyle changes with respect to the pre-pandemic period.

A general increase in unhealthy behavior was observed in the majority of the sample. As for the gender comparison, a higher proportion of women reported their diet and sleep worsening, a reduction in PA, and walking for both duties and pleasure than males.

As for correlations, female gender was found to be positively related to knowledge (tau-b = 0.138, *p* = 0.000) and weight change (tau-b = 0.073, *p* = 0.015), and negatively correlated with dietary habits (tau-b = −0.181, *p* = 0.000), general PA (tau-b = −0.268, *p* = 0.000), walking for duty (tau-b = −0.310, *p* = 0.000), walking for pleasure (tau-b = −0.211, *p* = 0.000), and sleep (tau-b = −0.426, *p* = 0.000). Age correlated negatively with the knowledge level (tau-b = −0.076, *p* = 0.002), and with dietary habits (tau-b = −0.207, *p* = 0.000), smoking habit (tau-b = −0.251, *p* = 0.000), general PA (tau-b = −0.241, *p* = 0.000), walking for duty (tau-b = −0.066, *p* = 0.008), and sleep (tau-b = −0.305, *p* = 0.000). A higher education was found to be related to knowledge (tau-b = 0.072, *p* = 0.009), walking for pleasure (tau-b = 0.071, *p* = 0.011), sleep (tau-b = 0.081, *p* = 0.005) and smoking (tau-b = 0.076, *p* = 0.008) changes. The place of residence positively correlated with weight change (tau-b = 0.060, *p* = 0.045) and negatively correlated with COVID-19 knowledge (tau-b = −0.152, *p* = 0.000), dietary habits (tau-b = −0.109, *p* = 0.000), smoking habits (tau-b = −0.118, *p* = 0.000) and sleep (tau-b = −0.142, *p* = 0.000).

## 4. Discussion

Older people are one of the main targets of COVID-19 and its complications. It is demonstrated that understanding older people’s knowledge, perceived beliefs, and behaviors toward COVID-19 can be a pivotal step in preventing the spread of COVID-19 in this population [28].

Our study found a satisfactory level of knowledge about COVID-19, and related control measures, in the sample of the Italian elderly examined. These data are in line with other studies performed in older people [28] and in the general population in China and the US [14,29], but higher than those reported in a sample of the Brazilian elderly affected by diabetes [30].

Nevertheless, considering that our study was performed at an advanced stage of the pandemic, a high level of general knowledge about the modes of transmission, which has been proved to be a salient factor in influencing the adoption of preventive measures, was expected among older people; however, more than 10% of our sample was not aware that the main transmission route is air and that the etiological agent is a virus. These findings confirms that older population should be further informed on the features of the disease due to their higher vulnerability [28]. In fact, it has been demonstrated that, in older populations, having correct knowledge is directly related to higher risk perception, adoption of correct practice preventive measures, and avoiding medical care [31,32]. Moreover, the younger population living in the same geographical area during the first wave of the pandemic, showed a good level of knowledge regarding the disease and its features [19].

On the contrary, the results regarding participants’ knowledge of control measures was encouraging: the most effective protective measures were correctly identified (handwashing, facial masks, avoid close contacts, and vaccine). This could be explained by the communication campaign about epidemic control measures taken by the government, but also by individual efforts and the willingness of people to prevent the spread of the disease [28]. This highlights the importance of accompanying the implementation of control measures at the community level with correct information strategies which should be considered when fighting pandemic emergencies [22]. Moreover, to ensure the effectiveness of warnings, messages issued by public health communicators should be comprehendible, concise, and convincing [31].

High tendencies to adopt these behaviors during the pandemic have been found in previous studies [28,33,34]. In particular, more than 80% of respondents reported increased handwashing, the wearing of face masks, and decreased close contact with other people, confirming that basic protective behaviors against COVID-19 have also been well understood among older people [28]. With regards to handwashing and the use of face masks, it should be noted that they had already proved to be efficacious in preventing the spread of other diseases such as influenza and SARS [35].

Previous research demonstrated that age was not a significant factor influencing preventive knowledge and behaviors, while another found that individuals aged 60 years or older, knew and implemented fewer preventive behavior changes compared to younger participants [34,36].

With regards to lifestyle behaviors, a worrying increase in unhealthy behaviors has been registered, as reported in previous studies and other age groups [19,20,37]. The majority of the participants modified their sleeping habits, PA, diet, and consequently their weight. In particular, they reported a decrease in PA and walking as a consequence of the pandemic emergency, even for the basic walking for duties. This is particularly important, especially when considering the role of PA as an immune function adjuvant to reduce the risk of communicable diseases [19]. As a matter of fact, scientific evidence has reported a dose–response relationship between PA performed before infection and a reduction in the incidence, duration, or severity of acute respiratory tract infections [38,39]. In the current situation, exercise can also mitigate the negative effects of isolation, such as stress, anxiety, and sedentarism, which further reduce immune function and increase the risk of developing or worsening, non-communicable diseases [40].

Also, the findings related to diet and weight should not be underestimated, especially because older people frequently suffer from underlying diseases, such as cardiometabolic diseases, whose management can be affected by dietary habits and fat accumulation [30]. With regards to weight, it should be noted that our sample was composed of a high proportion of overweight/obese individuals. This proportion is higher than that reported for the older Apulian population by the Italian surveillance system “Passi d’Argento” [41]. Considering the 66.3% of the sample declared an increase in weight during the pandemic, this result could actually be related to the adoption of unhealthy weight-related behaviors.

Finally, sleep worsening was also reported. Sleep problems represent a common consequence of the COVID-19 pandemic worldwide [42]. Considering the beneficial role of sleep on immunity and psychological wellbeing, public health policies should tackle this issue [43,44].

Even for smoke habits, despite the high percentage of individuals who did not smoke at all in our sample, it should be noted that roughly 40% of participants continued, or started, to smoke.

With regards to gender comparison, a significantly higher risk of unhealthy habits regarding diet, PA, and sleeping, was found in females compared to male respondents. However, weight and smoking increases were more represented in males. This result is in line with findings of Füzéki et al., according to which there is a higher chance that women will fail to comply with lifestyle recommendations during lockdown [20], even though the sample of this latter study showed a mean age of 43.1 ± 11.3 years old, and age was not associated with the level of PA.

The correlation analysis confirmed the association between female gender and unhealthy behaviors; these were also related to a higher age, lower level of education, and living in facilities for self-sufficient individuals. A higher level of knowledge about COVID-19 and preventive measures was related to the female gender, lower age, higher education, and living in a community. The association between healthy habits, a lower age, and a high educational level was also found in a previous study performed to assess the levels of PA among older adults from the same population [24]. The result, related to the place of residence, is of concern because it suggests that institutionalization may affect both the awareness of COVID-19 and the lifestyle of the elderly. Further research is needed to clarify this aspect.

As for the lifestyle changes that occurred, it should be noted that both global and national healthcare institutions launched several information campaigns since the first months of the pandemic, aimed at promoting the importance of maintaining healthy behaviors while staying at home [45,46]. However, adherence to these guidelines can be affected by many factors, including how these messages are communicated and the attitudes of the individuals. In this context, healthcare professionals are fundamental to convey the right messages, in the right way, to their patients.

### Limitations of the Study

The authors are aware of the limitations of this study. First of all, participants were enrolled by convenience sampling from a large region in southern Italy; thus, the generalizability of our findings may be limited. Moreover, the survey was performed via an online questionnaire. This methodology has some important strengths, such as easier questionnaire preparation and distribution, data collection, and storing and analyzing at a lower cost and in a shorter period [47]. Furthermore, this methodology was particularly useful during a pandemic when contact between people should be avoided. On the other side, among the main weaknesses related to this issue are problems related to the sampling selection, unavailable response rate, and information accuracy can be listed, especially in a sample of older adults [47]. Finally, behaviors related to the adoption of preventive measures have not been investigated to avoid an excessive length of the questionnaire.

However, this is the first study offering a picture of knowledge about COVID-19, related control measures, and the lifestyle behaviors adopted during the pandemic in a specific sample of elderly people from Italy. Moreover, the study enrolled a large number of people in a short period of time, while a wide debate on vaccination policies and mandatory use of vaccine passports is underway. Therefore, our results are of practical significance for the design and implementation of health programs for the older population, not only targeted to the implementation of the preventive measures against COVID-19, but also to contribute to their acceptance. With regards to lifestyle habits, few data are reported in scientific literature regarding this specific age group; nevertheless, our data demonstrate critical issues that must be addressed. Further studies are needed to confirm these data and deepen the related factors to plan effective health promotion interventions.

## 5. Conclusions

Older adults who participated in this study showed a good level of knowledge about COVID-19 characteristics and prevention. However, effective warnings and messages issued by public health communicators still seem to be necessary. In contrast, participants reported an increase in unhealthy habits, which may have important health consequences in the long term. This represents a further negative consequence of the pandemic and should be addressed by public health interventions targeted at older people. In this context, both healthcare and social institutions can play a fundamental role in the implementation of health promotion initiatives aimed at educating individuals towards the adoption of healthy lifestyles during the pandemic. At the same time, healthcare professionals, especially physicians, are fundamental in enhancing their patients’ participation and compliance to these interventions.

## Figures and Tables

**Table 1:** Socio-demographic, anthropometric and behavioral characteristics of participants.

Variable	Participants *n* = 1041
gender	
males	434 (41.7)
females	607 (58.3)
age (mean ± SD)	76.6 (6.5)
educational level	
elementary	28 (2.7)
middle	310 (29.8)
high school	515 (49.5)
degree	188 (18.1)
place of residence	
community	953 (91.5)
institution	88 (8.5)
BMI (kg/m^2^)	
underweight	4 (0.4)
normal weight	246 (23.6)
overweight	517 (49.7)
obese	274 (26.3)

BMI: Body Mass Index participants who reported an overweight condition were mainly females with a high educational level and living in a community setting.

**Table 2:** Answers related to participants’ knowledge about COVID-19.

Questions	Respondents *n* (%)
may the new epidemic be considered similar to that of the seasonal flu?	
Yes	167 (16)
No	874 (84)
how many Italian people died by COVID-19?	
1000	6 (0.6)
10,000	187 (17.9)
more than 100,000	848 (81.5)
the cause of COVID-19 is a:	
Virus	916 (88)
Bacterium	117 (11.2)
Parasite	8 (0.8)
which is the target population of COVID-19?	
only older people	148 (14.2)
only children	2 (0.2)
only health care personnel	1 (0.1)
All	890 (85.5)
the main transmission route of the disease is:	
Air	1024 (89.8)
Water	65 (5.7)
Food	23 (2.0)
blood transfusion	28 (2.5)
the most effective protective measure is: *	
Handwashing	994 (95.5)
facial masks	1019 (97.9)
protective glasses	140 (13.4)
fruit and vegetables washing	69 (6.6)
disinfection of surfaces	334 (32.1)
avoid close contacts	908 (87.2)
Vaccine	1008 (96.8)
is there an effective drug to treat COVID-19?	
Yes	260 (25)
No	781 (75)

* multiple answers allowed.

**Table 3:** Answers related to participants’ lifestyle during the COVID-19 pandemic.

Questions	Respondents *n* (%)			
	Whole Sample	Males	Females	*p*
weight				0.000
increased	690 (66.3)	316 (72.8)	374 (61.6)	
decreased	123 (11.8)	70 (16.1)	53 (8.7)	
same as before	228 (21.9)	48 (11.1)	180 (29.7)	
diet				0.000
improved	13 (1.2)	6 (1.4)	7 (1.2)	
worsened	661 (63.5)	230 (53)	431 (71)	
same as before	367 (35.3)	198 (45.6)	169 (27.8)	
smoking				0.000
less than before or stopped	91 (8.7)	55 (12.7)	36 (5.9)	
more than before or started	169 (16.2)	78 (18)	91 (15)	
smoking as before	251 (24.1)	131 (30.2)	120 (19.8)	
not smoking as before	530 (50.9)	170 (39.2)	360 (59.3)	
physical activity				0.000
increased	32 (3.1)	23 (5.3)	9 (1.5)	
decreased	606 (58.2)	185 (42.6)	421 (69.4)	
same as before	403 (38.7)	226 (52.1)	177 (29.2)	
walking for duties				0.000
increased	116 (11.1)	108 (24.9)	8 (1.3)	
decreased	625 (60)	194 (44.7)	431 (71)	
same as before	300 (28.8)	132 (30.4)	168 (27.7)	
walking for pleasure				0.000
increased	184 (17.7)	105 (24.2)	79 (13)	
decreased	569 (54.7)	181 (41.7)	388 (63.9)	
same as before	288 (27.7)	148 (34.1)	140 (23.1)	
sleep				0.000
improved	4 (0.4)	3 (0.7)	1 (0.2)	
worsened	665 (63.6)	172 (39.6)	493 (81.2)	
same as before	372 (35.7)	259 (59.7)	113 (18.6)	

## Data Availability

All data presented are available upon request from the corresponding author (F.G.).
